# Resting state amygdala-prefrontal connectivity predicts symptom change after cognitive behavioral therapy in generalized social anxiety disorder

**DOI:** 10.1186/s13587-014-0014-5

**Published:** 2014-12-09

**Authors:** Heide Klumpp, Michael K Keutmann, Daniel A Fitzgerald, Stewart A Shankman, K Luan Phan

**Affiliations:** Mood and Anxiety Disorders Research Program, Department of Psychiatry (HK, DAF, KLP), University of Illinois at Chicago, 1747 W. Roosevelt Rd, Chicago, IL 60608 USA; Department of Psychology (HK, MKK, SAS, KLP), University of Illinois at Chicago, Chicago, IL USA; Department of Psychiatry (HK, SAS, KLP), University of Illinois at Chicago, Chicago, IL USA; Mental Health Service (DAF, KLP), Jesse Brown VA Medical Center, Chicago, IL USA

**Keywords:** Generalized social anxiety, fMRI, Treatment, Brain imaging, Rest

## Abstract

**Background:**

Aberrant amygdala-prefrontal interactions at rest and during emotion processing are implicated in the pathophysiology of generalized social anxiety disorder (gSAD), a common disorder characterized by fears of potential scrutiny. Cognitive behavioral therapy (CBT) is first-line psychotherapy for gSAD and other anxiety disorders. While CBT is generally effective, there is a great deal of heterogeneity in treatment response. To date, predictors of success in CBT for gSAD include reduced amygdala reactivity and increased activity in prefrontal regulatory regions (e.g., anterior cingulate cortex, “ACC”) during emotion processing. However, studies have not examined whether tonic (i.e., at rest) coupling of amygdala and these prefrontal regions also predict response to CBT.

**Results:**

Twenty-one patients with gSAD participated in resting-state functional magnetic resonance imaging (fMRI) before 12 weeks of CBT. Overall, symptom severity was significantly reduced after completing CBT; however, the patients varied considerably in degree of symptom change. Whole-brain voxel-wise findings showed symptom improvement after CBT was predicted by greater right amygdala-pregenual ACC (“pgACC”) connectivity and greater left amygdala-pgACC coupling encompassing medial prefrontal cortex. In support of their predictive value, area under receiver operating characteristic curve was significant for the left and right amygdala-pgACC in relation to treatment responders.

**Conclusions:**

Improvement after CBT was predicted by enhanced resting-state bilateral amygdala-prefrontal coupling in gSAD. Preliminary results suggest baseline individual differences in a fundamental circuitry that may underlie emotion regulation contributed to variation in symptom change after CBT. Findings offer a new approach towards using a biological measure to foretell who will most likely benefit from CBT. In particular, the departure from neural predictors based on illness-relevant stimuli (e.g., socio-emotional stimuli in gSAD) permits the development of biomarkers that reflect commonalities in the neurobiology of anxiety and mood disorders.

## Background

Cognitive behavioral therapy (CBT) is empirically supported psychotherapy for generalized social anxiety disorder (gSAD), a common, debilitating illness marked by excessive fears of negative evaluation by others [1]. CBT primarily attempts to reduce symptoms via cognitive restructuring, an emotion regulation strategy aimed at decreasing thought-related negative affect, in conjunction with exposure exercises (e.g., facing anxiety-evoking situations). While generally effective, treatment response is varied with approximately 30%–40% of patients with gSAD not fully responding to CBT [2,3]. Findings from neuroimaging studies indicate heterogeneity in treatment outcome may relate in part to brain regions implicated in the pathophysiology of gSAD that are utilized by CBT.

Accumulating data indicate the amygdala, a key emotion processing region that mediates fear [4], plays a prominent role in gSAD. The amygdala has interconnections to prefrontal regions that down-regulate emotional reactivity (e.g., medial prefrontal cortex (mPFC); [5]). In gSAD, amygdala hyper-reactivity to salient signals has been demonstrated in addition to disturbances in regulatory regions (e.g., exaggerated or attenuated mPFC activation; [6]). Moreover, in the absence of stimuli presentation or task engagement (i.e., during rest), aberrant amygdala connectivity with prefrontal regulatory areas (e.g., anterior cingulate cortex (ACC), medial orbitofrontal cortex (mOFC)) has been observed [7-9]. Findings suggest phasic hyper-reactive amygdala responses to external information involve tonic disturbances in core amygdala-prefrontal circuitry [8] and that individual differences in such circuitry may factor into the likelihood of benefiting from CBT.

To date, studies of amygdala as a brain-based marker in predicting CBT response in gSAD appear to be limited to emotion perception tasks, and results have been mixed. For example, we recently showed less pre-CBT amygdala activity to emotional faces predicted CBT success [10]; however, other emotion processing studies of gSAD have not revealed amygdala effects [11,12]. Regarding prefrontal regions as predictors, we have observed a positive link between dorsal ACC and mOFC activity in gSAD during emotion processing and symptom improvement in CBT [10,12] even in the absence of amygdala findings [12]. However, it is not clear whether amygdala response contributed to symptom change as regions were examined in isolation as opposed to nodes in a network.

A means of increasing our understanding of amygdala-based circuitry as a biomarker in predicting who will likely respond to CBT is with resting-state functional MRI (rs-fMRI). An advantage of rs-fMRI is that it examines fundamental networks that are task independent but may underlie emotion and regulatory processes in the unprovoked state [13]. Therefore, the objective of this study was to use pre-CBT rs-fMRI to investigate the relationship between amygdala-prefrontal coupling and CBT success in gSAD. Based on the literature [10,12], we hypothesized greater rs-fMRI amygdala-ACC or amygdala-mOFC connectivity would correspond with CBT response.

## Methods

### Participants

All 21 participants (14 female, 7 male) with an average age of 28.3 ± 9.4 years met criteria for gSAD based on the Structured Clinical Interview for DSM-IV (SCID) [14]. Symptom severity was assessed with the Liebowitz Social Anxiety Scale (LSAS) [15] administered by licensed clinicians, and depression level was measured with the Beck Depression Inventory [16]. Clinical Global Impression-Improvement (CGI-I; [17]), comprising a 7-point scale (1 = very much improved, 7 = worsening symptoms), was used to determine whether or not a patient responded to treatment.

All the participants were free of psychotropic medication, except for two who were on a stable dose of bupropion for at least 8 weeks prior to, and throughout, the study. Exclusion criteria included current or recent (within 6 months of study) comorbid major depressive disorder or recent substance abuse/dependence or any history of major psychiatric illness (e.g., bipolar, psychotic disorder).

The participants were between 18 and 55 years of age, right-handed, and free of current and past major medical or neurologic illness, as confirmed by a Board Certified physician. None of the participants tested positive for alcohol or illegal substances. The study protocol was approved by the Institutional Review Boards of the University of Michigan Medical School, and as per protocol, all the participants provided written informed consent.

The patients received 12 weeks of manualized individual CBT conducted by the same doctoral-level licensed clinical psychologist who has several years of training in CBT. A licensed clinical psychologist with both expertise in CBT and clinical trial investigations involving CBT provided supervision to ensure adherence to treatment. CBT encompassed psychoeducation, cognitive restructuring, *in vivo* exposures, and relapse prevention [18].

### Resting-state fMRI

Padding with foam cushions was used to reduce head movement. The participants were instructed to fixate on a crosshair centrally displayed on the blank gray screen, relax, and let their mind wander without falling asleep for 8 min.

### Functional imaging: acquisition and analysis

Magnetic resonance imaging (MRI) was performed on a 3 T GE Signa System (Milwaukee, WI) acquiring blood-oxygen-level-dependent (BOLD) images with a T2*-sensitive gradient-echo reverse spiral acquisition (3 mm × 43 axial slices; 2 s TR; 30 ms TE; 64 × 64 matrix; 220 mm FOV; 90° flip) optimized to minimize susceptibility artifacts in the medial temporal pole. High-resolution, T1-weighted anatomical scans (3D-SPGR; 9 ms TR; 1.8 ms TE; 15° flip; 256 × 256 matrix; 256 mm FOV, 1.2 mm × 124 axial slices) were also acquired for precise anatomical localization and normalization.

Analyses were performed using the Functional Connectivity (CONN) toolbox [19], which employs routines from the Statistical Parametric Mapping software (SPM8; Wellcome Trust Centre for Neuroimaging, London, UK). Eight initial volumes from each resting-state run were discarded to allow for T1 equilibration effects. Images were realigned to correct for motion, corrected for errors in slice timing, spatially transformed to standard MNI space using the functional template provided with SPM8, resampled to 2-mm voxels, and smoothed with an 8-mm FWHM Gaussian kernel prior to statistical analysis. The participants had no movement greater than 2-mm translation or 2° rotation across the run. Effects of nuisance variables (global, white matter and CSF signals and movement parameters) were reduced following the CompCor strategy [20]; data were band-pass filtered to 0.01–0.09 Hz.

Temporal correlations of the resting-state BOLD signal time series were examined between the left and right amygdala “seed” regions (anatomically derived regions of interest from the Automated Anatomical Labeling (AAL) toolbox [21]) and the rest of the brain. During second-level processing, LSAS change (Δ_PreTx−PostTx_) was regressed with initial severity (LSAS_PreTx_) controlled for as a regressor of no interest. The ACC and medial OFC regions of interest were examined at the whole-brain level with significance defined as *p* < 0.005 uncorrected with more than 20 contiguous voxels per cluster (>160 volume mm^3^) to strike a balance between type I and II errors [22]. The AAL atlas [21,23] was used to identify regions of interest (ROIs) and other significant whole-brain findings across subjects.

To clarify the directionality and magnitude of baseline amygdala-prefrontal connectivity related to change in symptom severity, 10-mm-diameter spherical ROIs were generated around the peak activation of a whole-brain cluster. Subsequently, parameter estimates (β weights and arbitrary units (au)) were extracted from the ROIs for each participant and submitted to Pearson’s correlations and scatterplots in the Statistical Package for the Social Sciences (SPSS version 20; Chicago, IL). Additionally, the parameter estimates were used to calculate the area under a receiver operating characteristic (ROC) curve in SPSS to assess the predictive value of *a priori* connectivity results in terms of CBT responders based on CGI-I. Apart from fMRI, we performed a regression analysis in SPSS to examine whether demographic factors (i.e., age, gender, education level) independently effected LSAS change (Δ_PreTx−PostTx_).

## Results

### Treatment effects on social anxiety

Symptom severity assessed by LSAS significantly decreased from an average of 71.6 ± 11.9 to 51.5 ± 19.5 (*t* = 4.87, *p* < 0.001). The clinical cutoff is ≥60 for gSAD [24]; therefore, results point to a significant overall improvement with variation in degree of symptom change. Additionally, depression level which was in the minimal range [16] at the start of CBT (11.7 ± 8.3) significantly decreased (5.0 ± 6.0) (*t* = 4.60, *p* < 0.001). Based on the CGI-I, about 70% of the patients with gSAD (15 of 21) were “responders” as they were rated to be “very much improved” or “much improved” (CGI-I score of 1 or 2) whereas 6 patients had a CGI-I score of >2 post-treatment and were thus considered “non-responders.” Regression analysis findings were not significant for age, gender, or education level (all *ps* > 0.05).

### fMRI

**Figure 1:**
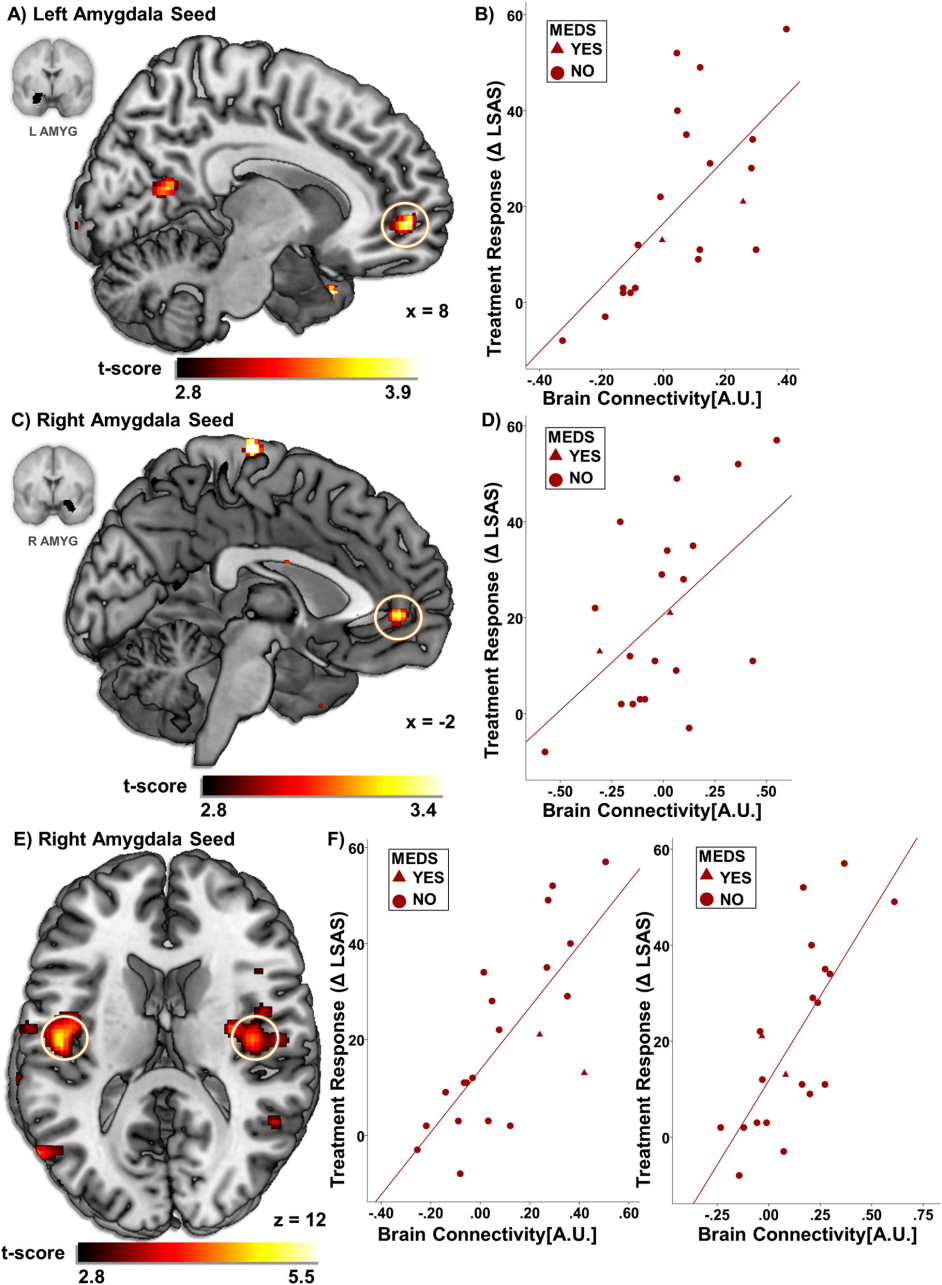
**Regressing LSAS change and scatterplot of regression analyses. (A)** Regressing LSAS change (Δ_PreTx**−**PostTx_) while initial severity (LSAS_PreTx_) is controlled for as a regressor of no interest; brain map depicts whole-brain analysis of covariance showing enhanced left amygdala-anterior cingulate cortex/medial prefrontal cortex coupling during rest in gSAD displayed on statistical t-map at *p* < 0.005. **(B)** Scatterplot of regression analyses depicting extracted measures of left amygdala-anterior cingulate cortex/medial prefrontal cortex connectivity and relation to change in social anxiety severity. **(C)** Regressing LSAS change (Δ_PreTx−PostTx_) while baseline severity (LSAS_PreTx_) is controlled for as a regressor of no interest; brain map depicts whole-brain analysis of covariance showing enhanced right amygdala-anterior cingulate cortex connectivity during rest in gSAD displayed on statistical t-map at *p* < 0.005. **(D)** Scatterplot of regression analyses depicting extracted measures of right amygdala-anterior cingulate cortex coupling and relation to change in social anxiety severity. **(E)** Regressing LSAS change (Δ_PreTx−PostTx_) while initial severity (LSAS_PreTx_) is controlled for as a regressor of no interest; brain map depicts whole-brain analysis of covariance showing enhanced right amygdala-bilateral insula connectivity during rest in gSAD displayed on statistical t-map at *p* < 0.005. **(F)** Scatterplot of regression analyses illustrating extracted measures of right amygdala-bilateral insula coupling and relation to change in social anxiety severity. LSAS, Liebowitz Social Anxiety Scale; CBT, cognitive behavioral therapy; gSAD, generalized social anxiety disorder.

For the right amygdala, LSAS change (Δ_PreTx−PostTx_) was predicted by more baseline connectivity with the left pregenual ACC (“pgACC”) (i.e., anterior cingulum) [(−4, 48, 0), *z* = 2.90, volume = 392 mm^3^; *r* = 0.55, *p* < 0.010] (Figure 1). Area under an ROC curve regarding the right amygdala-pgACC was 0.80 in the context of CBT responders which was significant (*p* < 0.04). Similar pgACC results were observed for the left amygdala [(10, 52, −2), *z* = 3.30, volume = 928 mm^3^; *r* = 0.66, *p* < 0.001] though here the cluster extended to the medial prefrontal cortex (i.e., frontal medial orbital gyrus) volume = 712 mm^3^ (Figure 1). Again, area under the curve (i.e., 0.83) was significant (*p* < 0.02). As to regions beyond *a priori* prefrontal areas, we observed symptom improvement robustly corresponded with bilateral insula (i.e., rolandic operculum) coupling [left: (−36, −30, 26), *z* = 4.00, volume = 2,648 mm^3^; *r* = 0.75, *p* < 0.001; right: (30, −10, 18), *z* = 4.14, volume = 2,192 mm^3^; *r* = 0.74, *p* < 0.001] related to the right amygdala. Area under the curves concerning the right amygdala-left insula and right amygdala-right insula were significant (i.e., 0.84, *p* < 0.02; 0.80, *p* < 0.04, respectively) (Figure 1). For completeness, we report all results outside regions of interest in Table 1.

**Table 1:** **Whole-brain voxel-wise regression: relation between pre-treatment to post-treatment change in social anxiety severity, controlling for pre-treatment severity**

		**MNI coordinates**			**Volume**	
	**Region**	***x***	***y***	***z***	**(mm** ^**3**^ **)**	***Z***
Right amygdala						
Positive correlation	R rolandic operculum	30	−10	18	2,192	4.14
	L rolandic operculum	−36	−30	26	2,648	4.00
	L middle temporal gyrus	−58	−66	12	800	3.67
	L angular gyrus	−36	−70	48	552	3.38
	R frontal inferior triangularis	48	26	10	760	3.26
	L middle temporal gyrus	−66	−34	2	608	3.18
	L paracentral lobule	−8	−20	78	344	3.15
	R middle frontal gyrus	26	34	34	408	2.96
	**L anterior cingulum**	−4	48	0	392	2.90
Negative correlation	L parahippocampal gyrus	−14	2	−18	528	4.01
	L temporal pole superior gyrus	−26	14	−26	440	4.00
	R hippocampus	24	−10	−20	328	3.98
	R frontal superior orbital gyrus	16	16	−18	264	3.73
	R gyrus rectus	6	32	−18	760	3.69
	R frontal superior gyrus	16	66	26	360	3.64
	R frontal middle orbital gyrus	38	42	−10	512	3.64
	R frontal superior medial gyrus	10	40	56	352	3.48
	L cerebellum	−4	−40	−18	336	3.33
	L frontal middle orbital gyrus	−22	58	−10	296	3.04
	L fusiform gyrus	−32	−18	−18	360	2.18
Left amygdala						
Positive correlation	L calcarine gyrus	−12	−60	16	1,136	4.44
	R temporal pole middle gyrus	32	18	−36	344	3.58
	**R anterior cingulum**	10	52	−2	928	3.30
	**R frontal medial orbital gyrus**				712	3.30
	L middle occipital gyrus	−38	−78	20	344	3.27
	R calcarine gyrus	8	−58	14	464	3.06
Negative correlation	L caudate	−18	−14	18	224	4.67
	R frontal middle orbital gyrus	36	64	−4	1,104	4.05
	L frontal middle gyrus	−44	20	48	1,104	3.97
	R inferior occipital gyrus	36	−64	−8	472	3.87
	L cerebellum	−42	−80	−22	784	3.77
	R frontal superior medial gyrus	6	42	52	1,744	3.74
	R frontal superior gyrus	14	68	24	576	3.68
	R cerebellum	4	−48	−44	432	3.60
	R frontal superior orbital gyrus	18	38	−16	376	3.53
	R frontal inferior orbital gyrus	36	38	−12	480	3.47
	L frontal middle orbital gyrus	−22	52	−10	360	3.36
	R superior parietal lobule	20	−62	60	440	3.21
	L cerebellum	−18	−90	−30	200	3.15
	R inferior temporal gyrus	56	−66	−8	296	3.00
	R frontal middle gyrus	42	16	54	168	2.84

All listed clusters are significant at *p* < 0.005 (uncorrected) with a threshold of greater than 160 volume (mm^3^).

Areas showing *a priori* hypothesized treatment-related predictors are bolded.

*MNI* Montreal Neurological Institute, *Z Z*-score.

## Discussion

As hypothesized, clinical improvement following CBT in the patients with gSAD was predicted by greater pre-treatment amygdala connectivity with prefrontal regions implicated in controlling emotion. Specifically, greater symptom reduction was foretold by increased pre-CBT right amygdala-pgACC and left amygdala-pgACC/mPFC coupling, a circuit involved in emotion processing and regulation [25,26]. In support of its predictive capacity, ROC results pertaining to CBT responder based on a CGI-I cutoff were also significant. Pointing to the potential relevance of the circuit as a brain predictor and/or target for treatment is a resting-state study showing lower amygdala-ACC/mPFC connectivity in gSAD correlated with social anxiety severity and that deficient coupling was enhanced by an acute challenge of the neuropeptide oxytocin [9]. Together, findings indicate intrinsic amygdala-medial prefrontal interactions may play a role in predicting the likelihood of responding to an intervention in gSAD. Findings expand on emotion activation paradigms that have demonstrated associations between increases in ACC or mOFC activity before treatment and improvement after CBT in gSAD [10,12]. Further study is needed to examine whether phasic (e.g., task/emotion-based) in combination with tonic (i.e., “at rest”) biomarkers can be used to predict response to CBT.

Beyond prefrontal regions of interest, symptom change was foretold by more and less connectivity in an extensive network indicative of the regions interconnected with the amygdala (e.g., insula, occipital lobe, middle temporal gyrus, superior frontal gyrus, parahippocampal gyrus; [27]) in addition to wide-scale coupling within and between networks exhibited at rest [13]. We did not have *a priori* hypotheses for these regions and, therefore, hesitate to interpret these preliminary, exploratory findings. Nevertheless, it is interesting to note symptom improvement also positively corresponded with the right amygdala-insula (i.e., rolandic operculum) coupling and, based on ROC findings, served as a good estimate of treatment response. The insula is proposed to play a role in anxiety disorders [28,29], which is supported by observations of exaggerated insula reactivity to emotional stimuli in gSAD relative to healthy controls [6]. In the context of treatment for gSAD, we observed insula hyper-reactivity to threat relevant stimuli decreased after CBT [12]; however, task-based pre-CBT insula activity to threat has not yet been shown to predict symptom change in gSAD [10-12]. Our findings suggest that in the absence of external stimuli, baseline emotion processing circuitry appears to function as a predictor. More study is needed to understand how the intrinsic amygdala-insula and other resting-state networks beyond *a priori* regions might be utilized by CBT.

## Conclusions

First, our study is not without important limitations. These include a relatively small sample size which increases risk for type II errors. Second, 2 of the 21 participants with gSAD were taking bupropion. Even though the medication was stable before the study and remained unchanged during the study, and these participants did not serve as outliers in *a priori* findings as indicated by scatterplots, any influence it may have had on other outcomes cannot be ruled out. Third, the lack of a waitlist group to serve as a control for changes in symptoms unrelated to treatment reduces our ability to draw firm conclusions about neural predictors of CBT response. Fourth, replication in an independent sample is necessary before conclusions can be made as to the clinical relevance of our findings. Fifth, connectivity results were limited to the bilateral amygdala. Future studies may want to “seed” prefrontal regions implicated in emotion regulation (e.g., dorsolateral, dorsomedial prefrontal cortex; orbitofrontal cortex; anterior cingulate cortex; [5]) to examine their relationship with the amygdala and ability to predict CBT success. Sixth, the lack of independent evaluators of treatment fidelity and symptom change warrants replication and further investigation. Despite limitations, findings suggest individual differences in intrinsic amygdala-prefrontal connectivity can help explain the heterogeneity in response to CBT in gSAD. Findings also indicate resting-state fMRI may be a useful approach in identifying brain-based biomarkers in treatment response. Among the advantages of resting-state biomarkers is the ease of application across other internalizing psychopathologies that may have common pathophysiology and for which CBT is an empirically validated treatment option (e.g., post-traumatic stress disorder, major depressive disorder).

## Abbreviations

CBT: cognitive behavioral therapy
gSAD: generalized social anxiety disorder
ACC: anterior cingulate cortex
mOFC: medial orbitofrontal cortex
rs-fMRI: resting-state functional magnetic resonance imaging
DSM-IV: diagnostic and statistical manual of mental disorders fourth edition
SCID: Structured Clinical Interview for DSM Disorders
LSAS: Liebowitz Social Anxiety Scale
BDI: Beck Depression Inventory
CGI-I: Clinical Global Impression-Improvement
BOLD: blood-oxygen-level-dependent
3T: 3.0 Tesla
GE: general electric
T2: spin-spin relaxation time
TR: repetition time
TE: echo time
ms: millisecond
mm: millimeter
FOV: field of view
3D-SPGR: three-dimensional spoiled gradient-recalled acquisition in steady state
FWHM: full width at half maximum
CSF: cerebrospinal fluid
CompCor: component-based noise correction method
Hz: Hertz
β: beta
pgACC: pregenual anterior cingulate cortex
mPFC: medial prefrontal cortex
PreTx: pre-treatment
PostTx: post-treatment
AAL: automated anatomical labeling
ROI: region of interest
SPSS: Statistical Package for the Social Sciences
ROC: receiver operating characteristic
au: arbitrary units
